# Outer membrane protein P4 is not required for virulence in the human challenge model of *Haemophilus ducreyi* infection

**DOI:** 10.1186/1471-2180-14-166

**Published:** 2014-06-24

**Authors:** Diane M Janowicz, Beth W Zwickl, Kate R Fortney, Barry P Katz, Margaret E Bauer

**Affiliations:** 1 Department of Medicine, Indiana University School of Medicine, 545 Barnhill Drive Room EH-435, Indianapolis, IN 46202, USA; 2 Department of Microbiology and Immunology, Indiana University School of Medicine, 635 Barnhill Drive Room MS-218, Indianapolis, IN 46202, USA; 3 Department of Biostatistics, Indiana University School of Medicine, 635 Barnhill Drive Room MS-218, Indianapolis, IN 46202, USA

**Keywords:** *H. ducreyi*, GUD, Chancroid, Lipoprotein, Human

## Abstract

**Background:**

Bacterial lipoproteins often play important roles in pathogenesis and can stimulate protective immune responses. Such lipoproteins are viable vaccine candidates. *Haemophilus ducreyi*, which causes the sexually transmitted disease chancroid, expresses a number of lipoproteins during human infection. One such lipoprotein, OmpP4, is homologous to the outer membrane lipoprotein *e* (P4) of *H. influenzae*. In *H. influenzae*, *e* (P4) stimulates production of bactericidal and protective antibodies and contributes to pathogenesis by facilitating acquisition of the essential nutrients heme and nicotinamide adenine dinucleotide (NAD). Here, we tested the hypothesis that, like its homolog, *H. ducreyi* OmpP4 contributes to virulence and stimulates production of bactericidal antibodies.

**Results:**

We determined that OmpP4 is broadly conserved among clinical isolates of *H. ducreyi*. We next constructed and characterized an isogenic *ompP4* mutant, designated 35000HP*ompP4*, in *H. ducreyi* strain 35000HP. To test whether OmpP4 was necessary for virulence in humans, eight healthy adults were experimentally infected. Each subject was inoculated with a fixed dose of 35000HP on one arm and three doses of 35000HP*ompP4* on the other arm. The overall parent and mutant pustule formation rates were 52.4% and 47.6%, respectively (*P =* 0.74). These results indicate that expression of OmpP4 in not necessary for *H. ducreyi* to initiate disease or progress to pustule formation in humans. Hyperimmune mouse serum raised against purified, recombinant OmpP4 did not promote bactericidal killing of 35000HP or phagocytosis by J774A.1 mouse macrophages in serum bactericidal and phagocytosis assays, respectively.

**Conclusions:**

Our data suggest that, unlike *e* (P4), *H. ducreyi* OmpP4 is not a suitable vaccine candidate. OmpP4 may be dispensable for virulence because of redundant mechanisms in *H. ducreyi* for heme acquisition and NAD utilization.

## Background

Bacterial genomes usually contain a significant portion of open reading frames (ORFs) that encode lipoproteins. For example, the genome of *Neisseria meningitidis* group B strain MC58 has 70 ORFs that encode surface-exposed or exported putative lipoproteins [1]. Approximately 8% of the ORFs of *Borrelia burgdorferi* encode putative lipoproteins [2]. The presence of numerous lipoproteins in bacterial genomes suggests their importance for bacterial survival and pathogenesis. Lipoproteins have been demonstrated to have roles in preserving membrane structure, functioning as enzymes, and serving as transporters or toxins. Lipoproteins also serve as immunogens; for example, the lipoprotein outer surface protein A (OspA), which plays important roles in *B. burgdorferi’s* biology, was used to develop an OspA-based vaccine [3,4].

*Haemophilus ducreyi*, the etiologic agent of the sexually transmitted genital ulcer disease chancroid, has the capacity to express 67 putative lipoproteins (GenBank accession number AE017143), only four of which have been well characterized: the peptidoglycan associated lipoprotein (PAL), the fibrinogen binding protein (FgbA), the *ducreyi* lectin A (DltA), and *H. ducreyi* lipoprotein (Hlp) [5-7]. PAL is conserved among *H. ducreyi* strains and contains a surface-exposed epitope defined by the monoclonal antibody 3B9 [8]. An isogenic PAL mutant is unable to cause pustules in the human infection model [9]. FgbA and DltA also contribute to *H. ducreyi* virulence in humans [5,10]. The roles of other lipoproteins in *H. ducreyi* pathogenesis have not yet been delineated.

In order to better understand the bacterial factors that contribute to the pathogenesis of *H. ducreyi,* an experimental human model of infection was developed [11,12]. In this model, adult volunteers are inoculated with *H. ducreyi* strain 35000HP, or its isogenic derivatives, on the skin overlying the upper deltoid. Within 24 h of inoculation, volunteers develop papules that either resolve or progress into pustules over the ensuing 2 weeks. Experimental infection mimics natural infection both clinically and histologically and has allowed identification of *H. ducreyi* genes that are expressed in vivo [13]. One of the genes identified as being expressed in multiple volunteers was *HD1170. HD1170* encodes a putative lipoprotein, designated outer membrane protein P4 (OmpP4).

OmpP4 is a homolog of the outer membrane lipoprotein *e* (P4) of *H. influenzae. e* (P4) is broadly conserved among typeable and nontypeable *H. influenzae* (NTHI) strains and is expressed as an abundant, immunodominant 28 kDa lipoprotein in outer membrane protein (OMP) fractions [14]. *e* (P4) was shown to play a role in virulence in an infant rat model of infection with *H. influenzae* type b [15]. Mechanistically, *e* (P4) is a phosphomonoesterase that facilitates the transport of two essential nutrients, heme and nicotinamide nucleotides, across the outer membrane of NTHI [16,17]. Monoclonal anti-*e* (P4) antibodies are highly reactive with a surface exposed epitope of *e* (P4), and anti-*e* (P4) serum is bactericidal against NTHI strains [14,18]. Immunization with *e* (P4) afforded protection against colonization in a mouse model of NTHI infection [19]. Thus, *e* (P4) is being actively investigated as a vaccine candidate against NTHI [18-20].

The predicted *H. ducreyi* OmpP4 shares 61% identity with *e* (P4), including conservation of the functional motifs required for enzymatic activity and for heme binding in *e* (P4) [21]. Because of its significant homology with *e* (P4) and its in vivo expression, we hypothesized that *H. ducreyi* OmpP4 may play an important role during human infection. Here, we found that *ompP4* is conserved among clinical isolates of *H. ducreyi*. To investigate its role in virulence and its utility as a vaccine candidate for *H. ducreyi*, we constructed and tested an isogenic o*mpP4* mutant in *H. ducreyi* 35000HP for virulence in human volunteers. We also tested whether mouse serum elicited against *H. ducreyi* OmpP4 promoted complement-mediated bactericidal activity or phagocytic uptake.

## Results

### Identification of the *ompP4* gene

**Figure 1:**
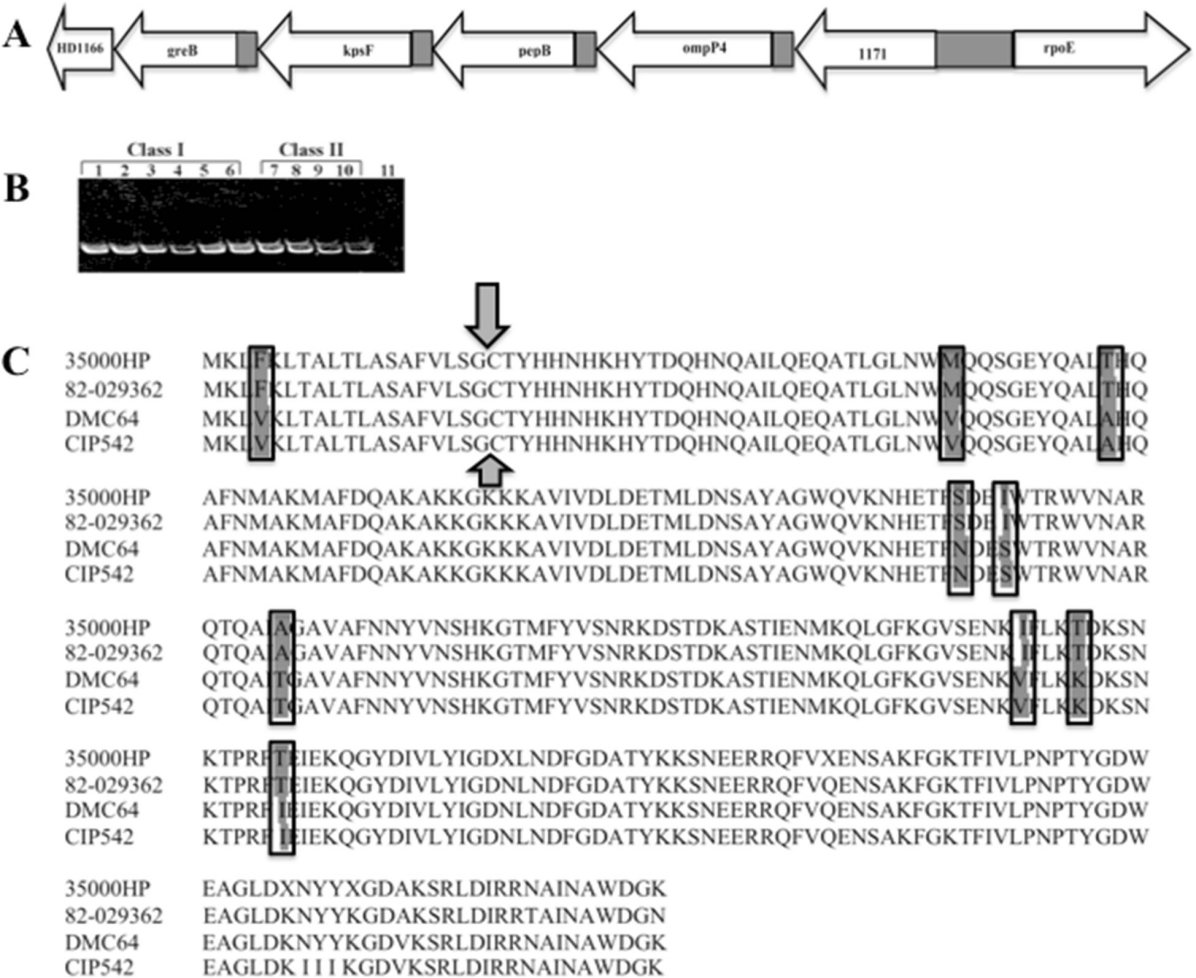
**Identification of the *ompP4 *gene within *H. ducreyi *35000HP. A**, Map of the *ompP4*–containing locus. **B**, PCR amplification of the *ompP4* locus from genomic DNA of ten clinical isolates. Lanes 1–6, class I strains 35000HP, HD183, HD188, 82–029362, 6644, and 85–023233, respectively; lanes 7–10, class II strains CIP542 TCC, DMC64, 33921 and HMC112, respectively; lane 11, negative control (no template added). **C**, Alignment of four deduced OmpP4 sequences among 2 class I strains (35000HP and 82–029362) and 2 class II strains (DMC64 and CIP542). Grey-highlighted residues are conserved within each class but differ between class I and class II strains. Shaded arrows denote the consensus signal peptide cleavage and lipidation site.

Analysis of the 35000HP genome identified an 831 bp open reading frame (ORF) that encoded an OmpP4 homologue. Sequence analysis of *ompP4* demonstrated an *N*-terminal signal II peptide and a consensus lipidation sequence, *N*-VLSGC-*C* (Figure 1). Based on sorting signals described for *Escherichia coli*, the presence of a tyrosine at position 2 suggests that OmpP4 sorts to the outer membrane [22,23]. The *ompP4* ORF lies within a putative operon (Figure 1). PCR amplification of the ORF of *ompP4* demonstrated that the gene was conserved in size and location among 10 different strains of *H. ducreyi* (Figure 1). Amplicons from two class I and two class II strains were sequenced and the deduced OmpP4 sequences compared. The deduced amino acid sequences and lengths of the ORFs were conserved within each class but differed by 9 amino acids between class I and class II strains (Figure 1).

### Construction and characterization of an *ompP4* mutant

**Figure 2:**
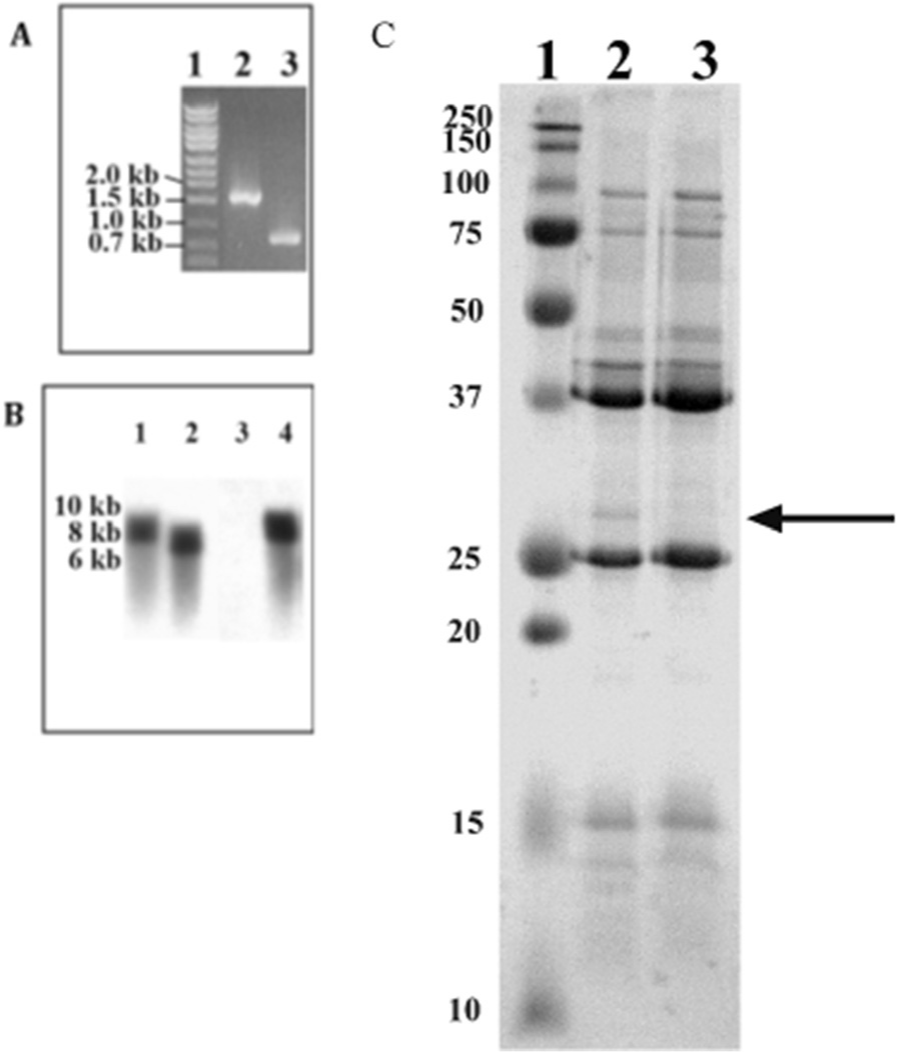
**Mutagenesis of *ompP4*. A**, Composite gel of the *ompP4* locus amplified using primers that flank the *ompP4* ORF. Lane 1, standard; lane 2, 35000HP*ompP4*; lane 3, 35000HP. **B**, Composite Southern blot of 35000HP*ompP4* and 35000HP probed with the cloned *ompP4* insert (lanes 1, 2) or the *kan* cassette (lanes 3, 4). Lanes 1 and 4, 35000HP*ompP4*; lanes 2 and 3, 35000HP. **C,** SDS-PAGE and Coomassie blue staining of OMPs prepared from 35000HP*ompP4* (lane 2) and 35000HP (lane 3); molecular markers are shown in lane 1, with sizes indicated to the left of the panel. Arrow points to the 30 kDa protein, the predicted size of OmpP4, missing in the *ompP4* mutant.

We constructed and characterized an isogenic *ompP4* mutant of *H. ducreyi* 35000HP, which was designated 35000HP*ompP4*. PCR amplification of the *ompP4* ORF in 35000HP*ompP4* demonstrated the size shift from 859 bp to 1.7 kb expected by addition of the 840 bp *kan* cassette (Figure 2A). In Southern blotting, the *kan* probe did not bind to the 35000HP genome but did bind to an 8.6-kb DNA fragment of the mutant genome, as expected. The *ompP4* probe bound to a 7.8-kb DNA fragment of the 35000HP genome and to an 8.6-kb fragment of the 35000HP*ompP4* genome (Figure 2B). Thus, the results from the PCR and Southern blot analyses were consistent with the insertion of a single antibiotic resistance cassette in the appropriate locus for the 35000HP*ompP4* mutant.

Sarkosyl insoluble membrane fractions were prepared from 35000HP*ompP4* and 35000HP. The fractions obtained from 35000HP*ompP4* were similar to those of 35000HP, except for lack of expression of a 30 kDa band (Figure 2C), the predicted size of OmpP4. These data suggest that OmpP4 does sort to the outer membrane [24]. 35000HP*ompP4* and 35000HP demonstrated similar lipooligosaccharide (LOS) profiles as analyzed by SDS-PAGE (data not shown). 35000HP*ompP4* and 35000HP demonstrated identical growth rates in broth (data not shown).

### Role of OmpP4 in experimental human infection

Eight healthy adults (three males, five females; 5 Caucasian, 3 black; age range 21 to 56; mean age ± standard deviation, 31 ± 11 years) volunteered for the study. One subject withdrew prior to inoculation. Two subjects (volunteers 313 and 314) were inoculated in the first iteration, two subjects (volunteers 316 and 317) in the second iteration, and three subjects (volunteers 324, 325, and 326) in the third iteration. An escalating dose–response study was used to compare the virulence of the mutant and the parent. In the first iteration, each subject was inoculated with a fixed estimated delivered dose (EDD) (143 CFU) of 35000HP at three sites on one arm and varying EDDs (51, 101 and 202 CFU) of 35000HP*ompP4* on the other arm (Table 1). Pustules formed at 2 of 6 parent sites and 5 of 6 mutant sites. Because the mutant was able to form pustules at doses similar to the parent, a second iteration using similar doses of parent and mutant was performed per protocol: 2 volunteers were inoculated with fixed EDD (128 CFU) of 35000HP on one arm and varying EDD (60, 119 and 238 CFU) of 35000HP*ompP4* on the other arm. Pustules formed at 5 of 6 parent sites and 5 of 6 mutant sites (Table 1). After two iterations, pustules formed at 7 of 12 parent sites and 10 of 12 mutant sites, suggesting that the mutant could be more virulent than the parent. As per protocol, an interim analysis was performed in order to determine the number of sites that needed to be inoculated with the mutant and the parent to have sufficient power to detect a difference in the pustule formation rate should 35000HP*ompP4* be more virulent than 35000HP. In the third iteration, 3 volunteers were inoculated with a parent dose (75 CFU) comparable to that of the mutant (116 CFU); pustules formed at 3 of 9 parent sites and at 1 of 9 mutant sites.

**Table 1:** **Response to inoculation of live *H. ducreyi *** **strains**

**Response to inoculation of live *H. ducreyi *** **strains**								
**Volunteer no.**	**Gender**^**a**^	**Days of observation**	**Isolate**^**b**^	**No. of initial Papules**	**No. of Pustules**	**Final outcome of sites**		
						**Papule**	**Pustule**	**Resolved**
313	F	7	P	3	2	1	2	
			M	3	3		3	
314	M	7	P	3	0			3
			M	3	2		2	1
316	F	7	P	3	3		3	
			M	3	3		3	
317	F	8	P	3	2		2	1
			M	3	2		2	1
324	M	8	P	3	1		1	2
			M	3	1		1	2
325	M	8	P	3	2		2	1
			M	3	0			3
326	F	6	P	3	0			3
			M	3	0			3

Volunteers 313 and 314 were inoculated in the first iteration. Volunteers 316 and 317 were inoculated in the second iteration. Volunteers 324, 325, and 326 were inoculated in the third iteration. ^a^F = female, M = male. ^b^P = 35000HP, M = 35000HP*ompP4*.

The overall papule formation rate for both the parent and the mutant was 100% at 21 sites each. Papules were similar in size at mutant sites (mean, 20.4 mm^2^) as at parent sites (mean, 27.6 mm^2^) 24 h after inoculation (*P =* 0.23). The overall pustule formation rate was 52.4% (95% CI, 23.3%-81.5%) at 21 parent sites and 47.6% (95% CI, 21.7%-73.5%) at 21 mutant sites (*P =* 0.74). Thus, the *ompP4* mutant was as virulent as the parent.

For the parent and mutant broth cultures used to prepare the inocula, 108 of 108 parent colonies and 107 of 107 mutant colonies tested were phenotypically correct (parent, kanamycin-sensitive; mutant, kanamycin-resistant). *H. ducreyi* was recovered intermittently from surface cultures of sites inoculated with the parent or mutant. Of the 21 sites that were inoculated with the parent, 7 (33.3%) yielded at least one positive surface culture, while 9 of 21 mutant sites (42.9%) yielded a positive surface culture (*P* = 0.43). All colonies obtained from surface cultures (n = 284 and n = 471) and biopsy specimens (n = 72 and n = 144) from parent sites and mutant sites, respectively, were phenotypically correct. Thus, all tested colonies from the inocula, surface cultures and biopsy specimens had the expected phenotype.

### Biological activity of anti-OmpP4 antiserum

**Figure 3:**
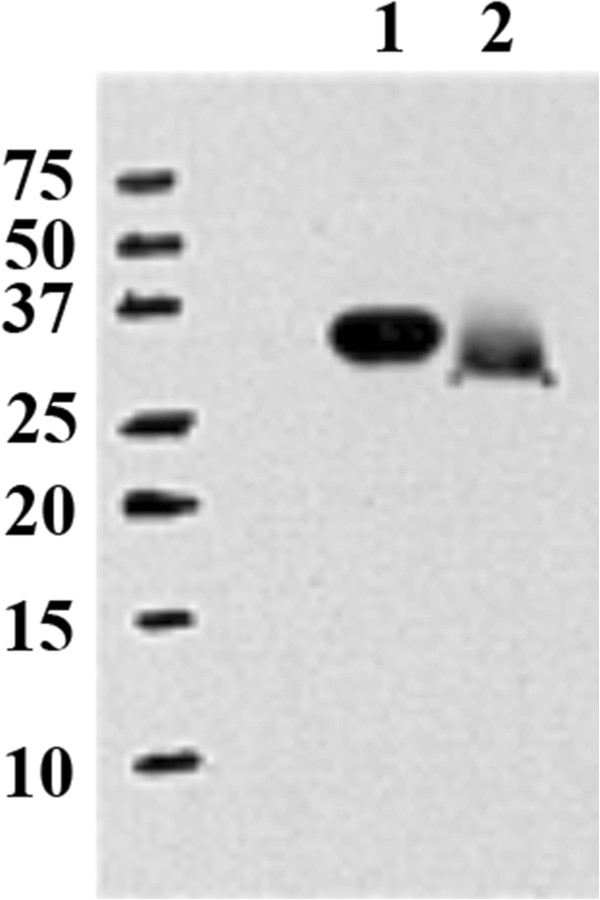
**Specificity of anti-OmpP4 antiserum.** Western blot probed with polyclonal antisera from mice inoculated with purified, recombinant OmpP4. Lane 1, purified recombinant OmpP4; lane 2, 35000HP whole cell lysates. The predicted molecular weight of recombinant, histidine-tagged OmpP4 is 29.2 kDa.

The abilities of *H. ducreyi* to resist phagocytosis and complement-mediated bactericidal activity are key features of the organism’s pathogenesis [10,25,26]. Although the *H. ducreyi ompP4* mutant was not attenuated for pustule formation in the human challenge model, immunization with OmpP4 could elicit protective antibodies that enhance bactericidal or phagocytic activity, as has been observed with NTHI *e* (P4). Therefore, we recombinantly expressed OmpP4 and tested its ability to generate biologically active antibodies in mice. Using Western blot analysis, the polyclonal mouse antiserum uniquely bound to purified recombinant OmpP4 and to a 29.2 kDa membrane protein, the predicted molecular weight of OmpP4, from whole cell lysates prepared from 35000HP (Figure 3)*.*

We used this hyperimmune mouse serum (HMS) raised against recombinant OmpP4 (HMS-P4) and compared the percent survival of 35000HP in 10% HMS-P4. As a positive control for bactericidal antibody activity against *H. ducreyi*, we used hyperimmune pig serum previously shown to enhance bactericidal activity (gift of Thomas Kawula) [27]. As expected, the mean percent survival of 35000HP decreased from 119.9% ± 41.4% in normal pig serum to 53.1% ± 12.4% in hyperimmune pig serum. In contrast, the mean percent survival of 35000HP was 63.0% ± 6.9% in normal mouse serum (NMS) compared with 93.4% ± 16.8% in HMS-P4. Thus, HMS-P4 did not promote bactericidal killing of 35000HP.

We next investigated the ability of HMS-P4 to promote phagocytosis of 35000HP by mouse monocyte-macrophage J774A.1 cells using quantitative phagocytosis assays. After opsonization with NMS, the mean percent phagocytosed 35000HP was 74.6% ± 11.5% compared to 86.3% ± 9.4% of bacteria phagocytosed after opsonization with HMS-P4 (P = 0.13); thus, anti-OmpP4 antibodies did not enhance phagocytosis of *H. ducreyi*.

## Discussion

*H. ducreyi* expresses at least 11 genes encoding lipoproteins in vivo [13], only three of which have been characterized for their roles in pathogenesis in humans [5,9,10]. For this study, we examined a previously uncharacterized lipoprotein, OmpP4, which has homology to the *H. influenzae* vaccine candidate *e* (P4). There are two phenotypic classes of *H. ducreyi* strains, which express different immunotypes and proteomes [28,29]. *ompP4* transcripts are expressed both in vitro and during human infection [13], and *ompP4* was conserved among all class I and class II clinical isolates of *H. ducreyi* that were tested, although there were minor differences in the deduced amino acid sequences between the class I and class II *ompP4* alleles sequenced. These data, coupled with the protein’s homology to *e* (P4), led us to hypothesize that OmpP4 may play an essential role in the formation of pustules in the human challenge model. However, 35000HP*ompP4* caused pustules to form at the same rate as the parent strain, indicating that *ompP4* is not necessary for virulence in humans. Whether o*mpP4* contributes to virulence for class II strains, which are not genetically tractable, is unknown.

The experimental model of human infection closely mimics natural infection, but it is limited to the papular and pustular stages of disease. In natural disease, pustules do not evolve into ulcers until several weeks after initial infection. Thus, we cannot rule out a role for OmpP4 during the ulcerative stage of disease. However, during experimental infection, *H. ducreyi* remains extracellular, where it associates with collagen, fibrin, polymorphonuclear leukocytes and macrophages. These relationships are maintained in natural ulcers [5] and thus it is unlikely that OmpP4 contributes to the ulcerative stage.

One of the attractive characteristics of *e* (P4) as a vaccine candidate is its ability to generate bactericidal and/or protective antibodies. We therefore examined whether antibodies against OmpP4 could block the organism’s ability to resist either serum bactericidal activity or phagocytosis. OmpP4-specific mouse antiserum had no effect on *H. ducreyi*’s survival in serum bactericidal assays or on *H. ducreyi*’s uptake by murine macrophages. It is possible that important conformational epitopes of native OmpP4 lipoprotein were not retained by the recombinant, non-lipidated OmpP4 antigen used. However, similar manipulations did not abrogate the ability of *e* (P4) to elicit bactericidal antibodies. Overall, our data suggest that, unlike NTHI *e* (P4), *H. ducreyi* OmpP4 is not a strong vaccine candidate.

*e* (P4) is essential for heme uptake by NTHI under aerobic conditions [15,16]. Like *H. influenzae*, *H. ducreyi* is dependent upon uptake of iron in the context of a porphyrin ring such as heme or hemoglobin for its survival. 35000HP*ompP4* and 35000HP had similar growth rates under the heme-replete conditions used for the human challenge model, suggesting that *ompP4* is not essential for heme uptake. *H. ducreyi* also encodes three TonB-dependent receptors, including hemoglobin receptor HgbA, heme receptor TdhA, and the uncharacterized TdX, for uptake of heme/iron. Among these three receptors, only HgbA is required for virulence in the human model of chancroid, and HgbA alone is both necessary and sufficient for heme/iron acquisition by *H. ducreyi*[30,31]. Thus, *H. ducreyi* expresses several redundant mechanisms for acquiring this essential nutrient, and any contribution of OmpP4 to heme/iron uptake, like those of TdhA or TdX, is likely secondary to the activity of HgbA.

*H. influenzae e* (P4) is necessary for utilization of the essential coenzyme NAD + (V factor). Members of the *Pasteurellaceae* cannot synthesize NAD + de novo and must salvage either NAD + or a suitable nicotinamide-based precursor from their environment [32]. So-called V-factor dependent *Pasteurellaceae* can only utilize NAD + or the precursors nicotinamide mononucleotide (NMN) or nicotinamide riboside (NR) [33,34]. This NAD + salvage pathway is well characterized in *H. influenzae*[32,34]: NAD+, NMN, and NR pass through porins into the periplasm, where NAD + is converted to NMN by the enzyme NadN, and NMN is converted to NR primarily through the catalytic activity of *e* (P4) [17,21,35]. The inner membrane transporter PnuC then transports NR into the cytoplasm, where the enzyme NadR converts NR to NAD + [36,37].

In contrast to *H. influenzae*, V-factor independent *Pasteurellaceae*, such as *H. ducreyi*, can utilize the precursor nicotinamide (NAm) to synthesize NAD + [34]. In this alternative salvage pathway, NAm diffuses across the cell wall into the cytoplasm, where the nicotinamide phosphoribosyltransferase NadV converts NAm to NMN, which is then converted to NAD + by an unidentified NMN adenylyltransferase [32,38]. Critical to this alternative salvage pathway is the enzyme NadV; in *H. ducreyi* strains, the *nadV* gene is carried on extrachromosomal or integrated copies of plasmid pNAD1, suggesting horizontal transfer of *nadV*[38,39]. Strain 35000HP, used to generate the *ompP4* mutant, contains two tandem, chromosomal copies of pNAD1 [39]. A previous study reported that *H. ducreyi* 35000HP encodes a complete *H. influenzae*-like NAD + salvage pathway [37]. However, at that time the *H. ducreyi* genome and its annotation were only available in preliminary form. Our analysis of the finalized *H. ducreyi* 35000HP genome showed that, while 35000HP includes full-length ORFs predicted to encode intact homologs of *e* (P4) (*ompP4*) and the NR transporter PnuC (*HD1041*), the homologs of *nadN* and *nadR* are pseudogenes. *H. influenzae* NadR is a bifunctional enzyme whose C-terminus contains NMN adenylyltransferase activity [37]. Possibly, the 3’ end of the *H. ducreyi nadR* pseudogene may express a truncated NadR with this activity. Alternatively, an as-yet-unidentified enzyme is required to convert NMN to NAD + in *H. ducreyi*. Overall, the absence of intact *nadN* and *nadR* genes suggests that the *H. influenzae*-like NAD + salvage pathway is dispensible in *H. ducreyi* because of NadV-driven utilization of NAm. NadV activity most likely accounts for our finding that 35000HP*ompP4* harbored no discernible growth defects. 35000HP is the only *H. ducreyi* strain whose genome is available to date; thus, whether OmpP4 activity is more critical for NAD + utilization in other *H. ducreyi* strains, and whether other strains harbor a complete *H. influenzae*-like NAD + salvage pathway, is unknown.

## Conclusions

The outer membrane protein OmpP4 is not required for virulence of *H. ducreyi* in human disease. Antibodies raised against the recombinant OmpP4 protein were not able to enhance phagocytic uptake or serum bactericidal activity, suggesting that OmpP4 would not be a suitable candidate for an *H. ducreyi* vaccine. The known functions of *e* (P4) in *H. influenzae*, including heme uptake and NMN conversion to NR in the NAD utilization pathway, are accomplished by different mechanisms in *H. ducreyi*. A common theme in bacterial pathogenesis is the redundancy of mechanisms used to accomplish tasks critical for a pathogen’s survival. Thus, although *e* (P4) plays an important role in *H. influenzae* pathogenesis, the activity of its homolog in *H. ducreyi* appears to be redundant with the virulence factor HgbA and the NadV-dependent NAD + salvage pathway.

## Methods

### Bacteria and culture conditions

35000HP is a human-passaged variant of strain 35000 and has been reported previously [40]. *H. ducreyi* strains were grown on chocolate agar plates supplemented with 1% IsoVitaleX at 33°C in 5% CO_2_ or in GC base broth culture supplemented with bovine hemin (50 mg/ml), 1% IsoVitaleX, and 5% fetal bovine serum.

### Conservation of *ompP4* in *H. ducreyi* clinical isolates

*H. ducreyi* strains have been categorized into one of two different classes, based on their OMP profiles and LOS migration patterns [5,28]. To examine whether *ompP4* was conserved among strains of both classes, we isolated genomic DNA from the following six class I strains: 35000HP (Winnipeg), HD183 (Singapore), HD188 (Kenya), 82–029362 (California), 6644 (Boston), and 85–023233 (New York). Genomic DNA was also isolated from the following four class II strains: CIP542 ATCC (Hanoi), HMC112 (CDC), 33921 (Kenya), DMC64 (Bangladesh). The *ompP4* ORF was PCR amplified, using primers 5’-GCGATATTAAGTGGCAACTAGCGG-3’ and 5’-GCAAATTAACCTCTCCCAACAGCCTG-3’ that were external to the ORF, from genomic DNAs isolated from the above strains. Amplicons from two class I and two class II strains were sequenced and compared.

### Construction and characterization of an *ompP4* mutant of strain 35000HP

An 840 bp *kan* cassette that consists almost entirely of *aphA-3* coding sequence from pUC18K3 [41] was ligated into a 3.9 kb *ompP4*-encoding region of the 35000HP genome that had been cloned into the pBluescript plasmid. Because o*mpP4* lies within a putative operon (Figure 1), a non-polar *kan* cassette was used, in which the 840 bp selectable kanamycin resistance gene (*aphA-3*) is immediately followed by a consensus ribosomal-binding site and a start codon [41].

Sequence analysis of one resulting plasmid confirmed that the *kan* cassette was inserted 187 bp downstream from the transcriptional start of the 831-bp *ompP4* ORF. A 4.7-kb EcoRI/XhoI fragment of this plasmid was subcloned into pRSM2072, which utilizes *lacZ* as a counter-selectable marker to facilitate allelic exchange [42]. The resulting plasmid was electroporated into strain 35000HP. Selection was performed on plates containing kanamycin (30 μg/mL). Colonies were then picked and grown on plates containing X-Gal (5-bromo-4-chloro-3-indoyl-β-D-galactopyranoside) and kanamycin. Cointegrates appeared as small blue colonies because the growth of 35000HP containing *lacZ* is suppressed in the presence of X-Gal [42]. *lacZ*-deficient colonies in which a second crossover event had occurred appeared as white, larger colonies. An *ompP4* mutant was recovered and designated 35000HP*ompP4*.

Construction of the mutant was confirmed using PCR amplification and Southern blotting. PCR amplification of the *ompP4* ORFs of 35000HP*ompP4* and 35000HP was performed using primers (5’-TGTACTTATCATCATAATCATAAGCAT-3’ and 5’-TTTGTTAGGATTAACTCGTTATTCA-3’) specific to the intergenic regions flanking *ompP4*, followed by agarose gel electrophoresis. For Southern blot analysis, *H. ducreyi* DNA was digested to completion with PstI, electrophoresed on 0.8% agarose gels and probed with either the cloned *ompP4* insert or the *kan* cassette.

LOS and OMPs were purified from 35000HP and 35000HP*ompP4* and analyzed by sodium dodecyl sulfate-polyacrylamide gel electrophoresis, as described [9]. The growth rates of parent and mutant in broth used to prepare the challenge inocula were also compared.

### Human inoculation protocol

Stocks of 35000HP and 35000HP*ompP4* were prepared according to the US Food and Drug Administration guidelines (BB-IND 13046). For the human inoculation protocol, healthy adult male and female volunteers over 21 years of age were recruited for the study. Subjects gave informed consent for participation and for human immunodeficiency virus (HIV) serology, in accordance with the human experimentation guidelines of the U.S. Department of Health and Human Services and the institutional ethics committee of Indiana University-Purdue University of Indianapolis. The experimental protocol, preparation and inoculation of the bacteria, calculation of the EDD, and clinical observations were all done exactly as described previously [12,43]. Subjects were observed until they reached clinical endpoint, which was defined as resolution of all sites, development of a pustule that was either painful or > 4 mm in diameter, or 14 days after inoculation. Subjects were then treated with one dose of oral ciprofloxacin as described [44].

Comparison of papule and pustule formation rates for the two strains was performed using a logistic regression model with generalized estimating equations (GEE) to account for the correlation among sites within the same individual, as previously described [43]. The GEE sandwich estimate for the standard errors was used to calculate 95% confidence intervals (95% CI) for these rates.

To confirm that the inocula contained or lacked the *kan* cassette and that the *kan* cassette was not lost by the mutant during the course of infection, individual colonies from the inocula, surface cultures and biopsy specimens were picked, suspended in freezing medium and frozen in 96-well plates. If available, thirty colonies from an individual specimen were scored for susceptibility to kanamycin on kanamycin-containing chocolate agar plates as described [31].

### Recombinant fusion protein construction and expression

The *ompP4* ORF, without the signal peptide sequence, was amplified from 35000HP genomic DNA using synthetic primers (5’-TGTACTTATCATCATAATCATAAGCAT-3’ and 5’-TGAATAACGAGTTAATCCTAACAAAA-3’) and then cloned into the pCR-XL-TOPO vector using the TOPO XL Cloning Kit (Invitrogen Corp, San Diego, Calif). The fragment was excised using EcoRI and then cloned into pRSETB (Invitrogen). Transformation of recombinant plasmid into BL21(DE3)pLysS cells allowed for fusion protein expression. Recombinant OmpP4 was expressed in inclusion bodies and was purified under conditions using urea following the QIAexpressionist System (Qiagen, Inc, Valencia, Calif). Stepwise dialysis with decreasing urea concentrations was used to remove urea from the recombinant proteins and then concentrated with a Centricon-10 microconcentrator (Amicon Corp., Beverly, Mass). Purified recombinant OmpP4 was used to inoculate BALB/c mice to produce polyclonal antibodies (Harlan Bioproducts for Science) that were used in bactericidal and phagocytosis assays.

### Immune serum bactericidal assays

35000HP was grown for 16–18 h from a freezer stock on chocolate agar plates at 33°C with 5% CO_2_ and harvested in phosphate-buffered saline. After vortexing for 30 sec, cells were suspended in GC medium and diluted to a final concentration of approximately 10^3^ to 10^4^ CFU/ml. Bactericidal assays were performed in 96-well plates. Each well received 50 μl 35000HP and 10 μl (or 10%) of heat-inactivated NMS or HMS-P4 and brought to 65 μl with GC broth. Plates were incubated for 30 min at 33°C with 5% CO_2_. Then, 25 μl of either active or heat-inactivated normal human serum, which was used as the complement source, was added and the plates were incubated for an additional 60 min at 33°C with 5% CO_2._ Bacteria were quantified by plating 100 μl from each well onto chocolate agar and incubating for 48 h at 33°C with 5% CO_2_. Heat-inactivated hyperimmune pig serum collected after multiple inoculations with *H. ducreyi*, which has been shown to promote bactericidal activity against *H. ducreyi*, was used as a positive control (kindly provided by Thomas Kawula, University of North Carolina, Chapel Hill) [27]. Data were reported as percent survival in active NHS compared to that in heat-inactivated-NHS. Each experiment was repeated three times, and arithmetic mean and standard deviation of the percent survival were calculated.

### Phagocytosis assays

The mouse monocyte-macrophage cell line J774A.1 (TIB-67; American Type Culture Collection) was cultured in Dulbecco's modified Eagle's medium (DMEM; BioWhittaker) supplemented with 4 mM GlutaMAX, 10% (vol/vol) heat-inactivated fetal bovine serum (FBS), and 1 mM sodium pyruvate. The cells, which were kept in culture for less than 1 month, were used only at low passage numbers. Twenty hours before infection, the cells were allowed to adhere to coverslips in 24-well tissue culture plates (2 × 10^5^ cells/well). The following day, nonadherent cells were removed by washing twice with RPMI-F. 35000HP containing the green fluorescent protein-expressing plasmid pRB157K (courtesy of R. J. Blick and E. J. Hansen) was grown to mid-logarithmic phase in Columbia broth without FBS and with streptomycin (100 μg/ml) and then centrifuged at 6,500 × g for 10 min. 35000HP(pRB157K) was suspended to an OD_660_ of 0.2, yielding approximately 10^7^ CFU/ml. A 900 μl portion of bacteria was opsonized with 100 μl of either NMS or HMS-P4 and incubated for 30 min at RT. The suspensions were subjected to centrifugation, and the resulting pellets were suspended in 900 μl of RPMI-F. Approximately 2 × 10^6^ CFU of opsonized bacteria were added to wells containing J774A.1 cells (2 × 10^5^ cells) for a multiplicity of infection of 10:1. Samples were centrifuged at 150 x g for 2 minutes, and phagocytosis was allowed to proceed at 37°C for 40 min. Phagocytosis was stopped by placing the tissue culture plate on ice. Cells were then fixed with 3.7% paraformaldehyde in PBS.

Phagocytosis was evaluated by confocal microscopy, as described previously [43]. Briefly, after washing in DMEM-FBS, samples were stained with affinity-purified rat anti-mouse CD45 monoclonal antibody (R&D Systems, Minneapolis, MN) followed by DyLight Fluor 649-conjugated goat anti-rat secondary antibody (Jackson ImmunoResearch Laboratories, West Grove, Pa.). Nuclei were visualized with Hoechst 33342. Samples were mounted onto slides with Vectashield mounting medium (Vector Laboratories) and examined under an Olympus FV1000-MPE confocal laser-scanning microscope. To assess whether bacteria were phagocytosed or remained extracellular, arbitrary fields in each sample were optically sectioned in 0.2 μm steps. The optical sections were stacked and animated using ImageJ software (Rasband, W.S., ImageJ, U. S. National Institutes of Health, Bethesda, Maryland, USA) to allow for examination of the relative positions of the bacteria and eukaryotic cells in three dimensions. Numbers of intracellular and extracellular bacteria were recorded to determine percent of bacteria phagocytosed, which was calculated as: (total number of intracellular bacteria/total number of bacteria) x 100. Three independent experiments were performed and the mean percent phagocytosed bacteria was calculated and compared between bacteria opsonized with NMS and bacteria opsonized with HMS-P4. Statistical analysis was performed using paired Student’s t tests.

## Abbreviations

EDD: Estimated delivered dose; HMS-P4: Hyperimmune mouse serum raised against recombinant *H. ducreyi* OmpP4; *kan* cassette: Nonpolar mutagenic cassette encoding kanamycin resistance gene; LOS: Lipooligosaccharide; NMS: Normal mouse serum; NTHI: Nontypeable *Haemophilus influenzae*; OMP(s): Outer membrane protein(s); ORF: Open reading frame.

## Competing interests

The authors declare that they have no competing interests.

## Authors’ contributions

DMJ conceived and designed the study, carried out the in vitro work, directed the human challenge studies, and drafted the manuscript. BWZ carried out the clinical component of the human challenge studies. KRF carried out the in vitro component of the human challenge studies and participated in the bactericidal assays. BPK provided statistical design and analysis for the human challenge studies and analysis for the in vitro assays. MEB participated in the phagocytosis assays and analysis of data and contributed to drafting the manuscript. All authors read and approved the final draft.

## References

[B1] Pizza M Scarlato V Masignani V Giuliani MM Arico B Comanducci M Jennings GT Baldi L Bartolini E Capecchi B Galeotti CL Luzzi E Manetti R Marchetti E Mora M Nuti S Ratti G Santini L Savino S Scarselli M Storni E Zuo P Broeker M Hundt E Knapp B Blair E Mason T Tettelin H Hood DW Jeffries AC Identification of vaccine candidates against serogroup B meningococcus by whole-genome sequencing Science 2000 287 5459 1816 1820 10710308 10.1126/science.287.5459.1816

[B2] Fraser CM Casjens S Huang WM Sutton GG Clayton R Lathigra R White O Ketchum KA Dodson R Hickey EK Gwinn M Dougherty B Tomb JF Fleischmann RD Richardson D Peterson J Kerlavage AR Quackenbush J Salzberg S Hanson M van Vugt R Palmer N Adams MD Gocayne J Weidman J Utterback T Watthey L McDonald L Artiach P Bowman C Genomic sequence of a Lyme disease spirochaete, *Borrelia burgdorferi* Nature 1997 390 6660 580 586 9403685 10.1038/37551

[B3] Sigal LH Zahradnik JM Lavin P Patella SJ Bryant G Haselby R Hilton E Kunkel M Adler-Klein D Doherty T Evans J Molloy PJ Seidner AL Sabetta JR Simon HJ Klempner MS Mays J Marks D Malawista SE A vaccine consisting of recombinant borrelia burgdorferi outer-surface protein a to prevent Lyme disease. Recombinant outer-surface protein a Lyme disease vaccine study consortium N Engl J Med 1998 339 4 216 222 9673299 10.1056/NEJM199807233390402

[B4] Steere AC Sikand VK Meurice F Parenti DL Fikrig E Schoen RT Nowakowski J Schmid CH Laukamp S Buscarino C Krause DS Vaccination against Lyme disease with recombinant borrelia burgdorferi outer-surface lipoprotein a with adjuvant. Lyme disease vaccine study group N Engl J Med 1998 339 4 209 215 9673298 10.1056/NEJM199807233390401

[B5] Bauer ME Townsend CA Doster RS Fortney KR Zwickl BW Katz BP Spinola SM Janowicz DM A fibrinogen-binding lipoprotein contributes to the virulence of *Haemophilus ducreyi* in humans J Infect Dis 2009 199 5 684 692 19199547 10.1086/596656 PMC2650842

[B6] Leduc I Richards P Davis C Schilling B Elkins C A novel lectin, DltA, is required for expression of a full serum resistance phenotype in *Haemophilus ducreyi* Infect Immun 2004 72 3418 3428 15155648 10.1128/IAI.72.6.3418-3428.2004 PMC415671

[B7] Hiltke TJ Campagnari AA Spinola SM Characterization of a novel lipoprotein expressed by *Haemophilus ducreyi* Infect Immun 1996 64 5047 5052 8945545 10.1128/iai.64.12.5047-5052.1996 PMC174487

[B8] Spinola SM Griffiths GE Bogdan JA Menegus MA Characterization of an 18,000 molecular-weight outer membrane protein of *Haemophilus ducreyi* that contains a conserved surface-exposed epitope Infect Immun 1992 60 385 391 1370430 10.1128/iai.60.2.385-391.1992 PMC257640

[B9] Fortney KR Young RS Bauer ME Katz BP Hood AF Munson RS Spinola SM Expression of peptidoglycan-associated lipoprotein is required for virulence in the human model of *Haemophilus ducreyi* infection Infect Immun 2000 68 11 6441 6448 11035757 10.1128/iai.68.11.6441-6448.2000 PMC97731

[B10] Janowicz DM Leduc I Fortney KR Katz BP Elkins C Spinola SM A DltA mutant of *Haemophilus ducreyi* is partially attenuated in its ability to cause pustules in human volunteers Infect Immun 2006 74 2 1394 1397 16428791 10.1128/IAI.74.2.1394-1397.2006 PMC1360367

[B11] Spinola SM Wild LM Apicella MA Gaspari AA Campagnari AA Experimental human infection with *Haemophilus ducreyi* J Infect Dis 1994 169 1146 1150 8169411 10.1093/infdis/169.5.1146

[B12] Janowicz DM Ofner S Katz BP Spinola SM Experimental infection of human volunteers with *Haemophilus ducreyi*: fifteen years of clinical data and experience J Infect Dis 2009 199 1671 1679 19432549 10.1086/598966 PMC2682218

[B13] Bauer ME Fortney KR Harrison A Janowicz DM Munson RS Jr Spinola SM Identification of *Haemophilus ducreyi* genes expressed during human infection Microbiology 2008 154 Pt 4 1152 1160 18375807 10.1099/mic.0.2007/013953-0 PMC2852322

[B14] Green BA Farley JE Quinn-Dey T Deich RA Zlotnick GW The *e* (P4) outer membrane protein of *Haemophilus influenzae*: biologic activity of anti-*e* serum and cloning and sequencing of the structural gene Infect Immun 1991 59 9 3191 3198 1715322 10.1128/iai.59.9.3191-3198.1991 PMC258152

[B15] Morton DJ Smith A VanWagoner TM Seale TW Whitby PW Stull TL Lipoprotein *e* (P4) of *Haemophilus influenzae*: role in heme utilization and pathogenesis Microbes Infect 2007 9 8 932 939 17548224 10.1016/j.micinf.2007.03.013 PMC1975679

[B16] Reidl J Mekalanos JJ Lipoprotein *e*(P4) is essential for hemin uptake by *Haemophilus influenzae* J Exp Med 1996 183 2 621 629 8627173 10.1084/jem.183.2.621 PMC2192447

[B17] Reidl J Schlor S Kraiss A Schmidt-Brauns J Kemmer G Soleva E NADP and NAD utilization in *Haemophilus influenzae* Mol Microbiol 2000 35 6 1573 1581 10760156 10.1046/j.1365-2958.2000.01829.x

[B18] Mason KW Zhu D Scheuer CA McMichael JC Zlotnick GW Green BA Reduction of nasal colonization of nontypeable *Haemophilus influenzae* following intranasal immunization with rLP4/rLP6/UspA2 proteins combined with aqueous formulation of RC529 Vaccine 2004 22 25–26 3449 3456 15308371 10.1016/j.vaccine.2004.02.027

[B19] Hotomi M Ikeda Y Suzumoto M Yamauchi K Green BA Zlotnick G Billal DS Shimada J Fujihara K Yamanaka N A recombinant P4 protein of *Haemophilus influenzae* induces specific immune responses biologically active against nasopharyngeal colonization in mice after intranasal immunization Vaccine 2005 23 10 1294 1300 15652672 10.1016/j.vaccine.2004.08.042

[B20] Green BA Baranyi E Reilly TJ Smith AL Zlotnick GW Certain site-directed, nonenzymatically active mutants of the *Haemophilus influenzae* P4 lipoprotein are able to elicit bactericidal antibodies Infect Immun 2005 73 7 4454 4457 15972549 10.1128/IAI.73.7.4454-4457.2005 PMC1168610

[B21] Kemmer G Reilly TJ Schmidt-Brauns J Zlotnik GW Green BA Fiske MJ Herbert M Kraiss A Schlör S Smith A Reidl J NadN and *e* (P4) are essential for utilization of NAD and nicotinamide mononucleotide but not nicotinamide riboside in *Haemophilus influenzae* J Bacteriol 2001 183 13 3974 3981 11395461 10.1128/JB.183.13.3974-3981.2001 PMC95280

[B22] Yamaguchi K Yu F Inouye M A single amino acid determinant of the membrane localization of lipoproteins in *E. coli* Cell 1988 53 3 423 432 3284654 10.1016/0092-8674(88)90162-6

[B23] Tokuda H Matsuyama S Sorting of lipoproteins to the outer membrane in *E. coli* Biochim Biophys Acta 2004 1694 1–3 IN1 9 15672528

[B24] Spinola SM Peacock J Denny FW Smith DL Cannon JG Epidemiology of colonization by nontypable *Haemophilus influenzae* in children: a longitudinal study J Infect Dis 1986 154 100 109 3486923 10.1093/infdis/154.1.100

[B25] Bong CTH Throm RE Fortney KR Katz BP Hood AF Elkins C Spinola SM A DsrA-deficient mutant of *Haemophilus ducreyi* is impaired in its ability to infect human volunteers Infect Immun 2001 69 1488 1491 11179317 10.1128/IAI.69.3.1488-1491.2001 PMC98046

[B26] Janowicz DM Fortney KR Katz BP Latimer JL Deng K Hansen EJ Spinola SM Expression of the LspA1 and LspA2 proteins by *Haemophilus ducreyi* is required for virulence in human volunteers Infect Immun 2004 72 4528 4533 15271912 10.1128/IAI.72.8.4528-4533.2004 PMC470669

[B27] Cole LE Toffer KL Fulcher RA San Mateo LR Orndorff PE Kawula TH A humoral immune response confers protection against *Haemophilus ducreyi* infection Infect Immun 2003 71 6971 6977 14638786 10.1128/IAI.71.12.6971-6977.2003 PMC308944

[B28] White CD Leduc I Olsen B Jeter C Harris C Elkins C *Haemophilus ducreyi* outer membrane determinants, including DsrA, define two clonal populations Infect Immun 2005 73 4 2387 2399 15784585 10.1128/IAI.73.4.2387-2399.2005 PMC1087395

[B29] Post DM Gibson BW Proposed second class of *Haemophilus ducreyi* strains show altered protein and lipooligosaccharide profiles Proteomics 2007 7 3131 3142 17676659 10.1002/pmic.200600830

[B30] Leduc I Banks KE Fortney KR Patterson KB Billings SD Katz BP Spinola SM Elkins C Evaluation of the repertoire of the TonB-dependent receptors of *Haemophilus ducreyi* for their role in virulence in humans J Infect Dis 2008 197 1103 1109 18462159 10.1086/586901

[B31] Al-Tawfiq JA Fortney KR Katz BP Elkins C Spinola SM An isogenic hemoglobin receptor-deficient mutant of *Haemophilus ducreyi* is attenuated in the human model of experimental infection J Infect Dis 2000 181 1049 1054 10720530 10.1086/315309

[B32] Gazzaniga F Stebbins R Chang SZ McPeek MA Brenner C Microbial NAD metabolism: lessons from comparative genomics Microbiol Mol Biol Rev 2009 73 3 529 541 Table of Contents 19721089 10.1128/MMBR.00042-08 PMC2738131

[B33] Niven DF O'Reilly T Significance of V-factor dependency in the taxonomy of *Haemophilus* species and related organisms Int J Syst Bacteriol 1990 40 1 1 4 2145965 10.1099/00207713-40-1-1

[B34] Gerlach G Reidl J NAD + utilization in *Pasteurellaceae*: simplification of a complex pathway J Bacteriol 2006 188 19 6719 6727 16980474 10.1128/JB.00432-06 PMC1595515

[B35] Singh H Schuermann JP Reilly TJ Calcutt MJ Tanner JJ Recognition of nucleoside monophosphate substrates by *Haemophilus influenzae* class C acid phosphatase J Mol Biol 2010 404 4 639 649 20934434 10.1016/j.jmb.2010.09.065 PMC2992459

[B36] Sauer E Merdanovic M Mortimer AP Bringmann G Reidl J PnuC and the utilization of the nicotinamide riboside analog 3-aminopyridine in *Haemophilus influenzae* Antimicrob Agents Chemother 2004 48 12 4532 4541 15561822 10.1128/AAC.48.12.4532-4541.2004 PMC529221

[B37] Kurnasov OV Polanuyer BM Ananta S Sloutsky R Tam A Gerdes SY Osterman AL Ribosylnicotinamide kinase domain of NadR protein: identification and implications in NAD biosynthesis J Bacteriol 2002 184 24 6906 6917 12446641 10.1128/JB.184.24.6906-6917.2002 PMC135457

[B38] Martin PR Shea RJ Mulks MH Identification of a plasmid-encoded gene from *Haemophilus ducreyi* which confers NAD independence J Bacteriol 2001 183 4 1168 1174 11157928 10.1128/JB.183.4.1168-1174.2001 PMC94989

[B39] Munson RS Jr Zhong H Mungur R Ray WC Shea RJ Mahairas GG Mulks MH *Haemophilus ducreyi* strain ATCC 27722 contains a genetic element with homology to the vibrio RS1 element that can replicate as a plasmid and confer NAD independence on *Haemophilus influenzae* Infect Immun 2004 72 1143 1146 14742562 10.1128/IAI.72.2.1143-1146.2004 PMC321595

[B40] Al-Tawfiq JA Thornton AC Katz BP Fortney KR Todd KD Hood AF Spinola SM Standardization of the experimental model of *Haemophilus ducreyi* infection in human subjects J Infect Dis 1998 178 1684 1687 9815220 10.1086/314483

[B41] Menard R Sansonetti PJ Parsot C Nonpolar mutagenesis of the *ipa* genes defines IpaB, IpaC, and IpaD as effectors of *Shigella flexneri* entry into epithelial cells J Bacteriol 1993 175 5899 5906 8376337 10.1128/jb.175.18.5899-5906.1993 PMC206670

[B42] Bozue JA Tarantino L Munson RS Jr Facile construction of mutations in *Haemophilus ducreyi* using *lacz* as a counter-selectable marker FEMS Microbiol Lett 1998 164 269 273 9682476 10.1111/j.1574-6968.1998.tb13097.x

[B43] Spinola SM Bong CT Faber AL Fortney KR Bennett SL Townsend CA Zwickl BE Billings SD Humphreys TL Bauer ME Katz BP Differences in host susceptibility to disease progression in the human challenge model of *Haemophilus ducreyi* infection Infect Immun 2003 71 11 6658 6663 14573692 10.1128/IAI.71.11.6658-6663.2003 PMC219599

[B44] Banks KE Fortney KR Baker B Billings SD Katz BP Munson RS Jr Spinola SM The enterobacterial common antigen-like gene cluster of *Haemophilus ducreyi* contributes to virulence in humans J Infect Dis 2008 197 1531 1536 18422457 10.1086/588001

